# Blinding of study statisticians in clinical trials: a qualitative study in UK clinical trials units

**DOI:** 10.1186/s13063-022-06481-9

**Published:** 2022-06-27

**Authors:** Mais Iflaifel, Christopher Partlett, Jennifer Bell, Andrew Cook, Carrol Gamble, Steven Julious, Edmund Juszczak, Louise Linsell, Alan Montgomery, Kirsty Sprange

**Affiliations:** 1 Nottingham Clinical Trials Unit, University of Nottingham, Nottingham, UK; 2 National Perinatal Epidemiology Unit, Nuffield Department of Population Health, University of Oxford, Oxford, UK; 3 University of Southampton, Southampton, UK; 4 Liverpool Clinical Trials Centre, University of Liverpool, Liverpool, UK; 5 School of Health and Related Research, University of Sheffield, Sheffield, UK

**Keywords:** Blinding, Statisticians, Qualitative, Focus groups, Clinical trials, Clinical trials unit

## Abstract

**Background:**

Blinding is an established approach in clinical trials which aims to minimise the risk of performance and detection bias. There is little empirical evidence to guide UK clinical trials units (CTUs) about the practice of blinding statisticians. Guidelines recommend that statisticians remain blinded to allocation prior to the final analysis. As these guidelines are not based on empirical evidence, this study undertook a qualitative investigation relating to when and how statisticians should be blinded in clinical trials.

**Methods:**

Data were collected through online focus groups with various stakeholders who work in the delivery and oversight of clinical trials. Recordings of the focus groups were transcribed verbatim and thematic analysis was used to analyse the transcripts.

**Results:**

Thirty-seven participants from 19 CTUs participated in one of six focus groups. Four main themes were identified, namely statistical models of work, factors affecting the decision to blind statisticians, benefits of blinding/not blinding statisticians and practicalities. Factors influencing the decision to blind the statistician included available resources, study design and types of intervention and outcomes and analysis.

Although blinding of the statistician is perceived as a desirable mitigation against bias, there was uncertainty about the extent to which an unblinded statistician might impart bias. Instead, in most cases, the insight that the statistician offers was deemed more important to delivery of a trial than the risk of bias they may introduce if unblinded.

Blinding of statisticians was only considered achievable with the appropriate resource and staffing, which were not always available. In many cases, a standard approach to blinding was therefore considered unrealistic and impractical; hence the need for a proportionate risk assessment approach identifying possible mitigations.

**Conclusions:**

There was wide variation in practice between UK CTUs regarding the blinding of trial statisticians. A risk assessment approach would enable CTUs to identify risks associated with unblinded statisticians conducting the final analysis and alternative mitigation strategies. The findings of this study will be used to design guidance and a tool to support this risk assessment process.

**Supplementary Information:**

The online version contains supplementary material available at 10.1186/s13063-022-06481-9.

## Background

Blinding (also called masking) of group allocation from individuals involved in a research study [1] is an established methodology that is considered important in the conduct of randomised clinical trials (RCTs). The rationale for keeping clinicians, participants and outcome assessors blinded to treatment allocation has been extensively discussed and focuses on minimising the likelihood of differential treatment or assessments of outcomes [2, 3]. Studies aiming to quantify the impact of lack of blinding have reported exaggeration of treatment effects of up to 68% [4, 5]. However, there is literature that has challenged the dogma that blinding is always necessary and highlighted some challenges that might arise from using it [4, 6].

It is necessary to unblind statisticians to group allocation for the purposes of performing disaggregate analyses; however, this could happen at different timepoints during a trial. For example, statisticians might remain blinded until the final analysis. Alternatively, they might be unblinded at an earlier stage for the purposes of interim or safety analysis.

The definition of blinding within the Medicines and Healthcare products Regulatory Agency’s (MHRA) Good Clinical Practice is given as

“A procedure in which one or more parties to the trial are kept unaware of the treatment assignment(s). Single-blinding usually refers to the subject(s) being unaware, and double-blinding usually refers to the subject(s), investigator(s), monitor, and, in some cases, data analyst(s) being unaware of the treatment assignment(s)” [7].

Notably, this definition implies that blinding applies less frequently to data analysts or statisticians. The potential for the risk of bias arising from the statistician performing the analysis of the interim data and the final analysis has received little attention.

Several studies have reported that bias may be introduced by statisticians through various routes, e.g. when determining membership of analysis populations, influencing decisions related to the trial protocol, or through the selective use and reporting of statistical tests. Blinding the statistician until the analysis has been specified is one way to mitigate against this [1, 8]. Existing guidelines recommend blinding the statisticians before the final database lock [9], but these guidelines do not consider the trial-specific risk of blinding or not blinding the statistician.

Given that there is no available guidance for a risk-proportionate approach to blind trial statisticians in RCTs, it makes sense to attempt to understand current practice (Work as Done) within context [10]. In order to do this, it is important to gather a clear understanding of how blinding of trial statisticians is done by engaging various stakeholders who have experience in the delivery and oversight of RCTs.

This study is part of a wider project, Blinding of Trial Statisticians (BOTS), that aims to develop evidence-based (and risk-proportionate) guidance and recommendations on when and how to blind statisticians in clinical trials, using a mixed-methods approach. The aims of this qualitative study were t:o understand the various models of statistical work in clinical trials units (CTUs); the rationale, from various perspectives, of blinding or not blinding statisticians; the benefits and challenges of blinding and not blinding and finally, to present recommendations for improving practice in regard to blinding statisticians in RCTs.

## Methods

Qualitative focus groups (FG) were conducted by researchers (CP, KS and MI) at the University of Nottingham, Nottingham Clinical Trials Unit, United Kingdom (UK). The qualitative sub-study was undertaken between April and June 2021. The BOTS study received University of Nottingham Ethical approval (FMHS 213-0321) on 26 April 2021.

### Sample

A purposive sample of statisticians, trial managers, data managers, programmers and data coordinators were recruited via UK Clinical Research Collaboration (UKCRC) working groups, the National Institute for Health Research (NIHR) statistics group, Medical Research Council (MRC)-NIHR Trials Methodology Research Partnership (TMRP) working groups and UK Trial Managers’ Network (UKTMN). An invitation email was sent to all these specific groups to share it with all potential stakeholders. All stakeholders who responded with interest to participate where included in the focus groups.

### Recruitment and data collection

The research team approached potential participants for focus groups by sending them invitation emails including a participant information sheet (see Additional file 1) outlining the purpose of the study, and a consent form (see Additional file 2). The research team contacted the relevant organisation administrators to share the study invitation email with their members.

The researchers conducted focus groups through the Microsoft Teams platform. Focus groups were video and audio recorded, with consent, for later transcription and analysis. A topic guide was developed to help focus discussion [11] and was modified after piloting with the first focus group (see Additional file 3). Field notes were also used to record discussions and agreement during FGs.

### Analysis

MI and KS transcribed the audio-recorded data for the FGs. An inductive/deductive thematic approach [12] was used to identify participants’ perspectives regarding statisticians’ blinding in RCTs.

The two researchers (MI and KS) independently conducted initial open coding and categorisation with the aid of NVivo12, a qualitative data management software tool. The researchers anonymised participants’ information by using non-identifiable codes and removing identifying information. Differences in interpretation were resolved through discussion between coders and when required a third person from the research team (CP) was involved to help to increase the reliability of the research [13]. Categories and themes were developed by constantly refining the coding scheme and master themes were then identified. This study followed the consolidated criteria for reporting qualitative research (COREQ) guidelines [14] (see Additional file 4).

## Results

Six FGs were conducted. Thirty-seven participants from 19 out of 52 CTUs in England, Wales and Scotland, volunteered to participate. The pilot group was held with internal Nottingham CTU (NCTU) staff including a mixture of statisticians, trial managers, data managers and one data coordinator. A further five FGs were then conducted (two were statistician-focused and three were a mixture of trial managers, programmers, data managers and statisticians). Table 1 summarises the number and roles of each FG’s participants.

**Table 1:** Focus group participant characteristics

Professional role	FG 1 (*n* = 10)	FG 2 (*n* = 6)	FG 3 (*n* = 5)	FG 4 (*n* = 6)	FG 5 (*n* = 5)	FG 6 (*n* = 5)
	Mixed (NCTU only)	Statisticians		Mixed		
Statistician	3	6	5	4	1	3
Trial manager	3				2	1
Data manager	3			1		
Data coordinator	1				1	
Programmer				1	1	1

Four broad themes emerged from the analysis of the FG transcripts:

1. Statistical models of work
2. Factors affecting the decision to blind/not blind statisticians
3. Benefits of blinding/not blinding trial statisticians (TSs)
4. Practicalities

### Theme 1—Statistical models of work

Six models of working were identified after analysing the FGs transcripts and these are presented in Table 2. All models shared involved at least two statisticians, one typically more junior TS and one more senior (lead/principal statistician). In some cases, tasks were delegated to another statistician. In one model an additional non-blinded statistician was also involved in the delivery of the trial. In another model coded allocation groups were used to facilitate analysis and reporting of data split by treatment group without directly revealing participants’ allocated treatment. However, the utility and validity of such blinding has been questioned [15].

**Table 2:** CTU statistician roles and models of working

**Overview of statistician roles**					
**Role name**	**Description**				
Trial statistician	Typically responsible for day-to-day statistical input into the design, conduct and reporting of the trial. Roles include (but are not limited to): • Input into database and CRF design • Producing regular monitoring reports and liaising with trial committees (e.g. TMGs, DMCs and TSC) • Planning and conducting the interim (if applicable) and final statistical analysis				
Lead statistician^a^	Leads on and has overall responsibility for statistical aspects of the trial. Responsible for oversight of the TS including advice and quality control. Often a co-applicant involved in the grant funding application				
Non-blinded Statistician	A statistician (not the trial or lead) statistician that is able to access data by allocation. The main purpose of an unblinded statistician is to enable trial and lead statisticians to remain blinded where possible				
**Statistical models**					
**Role name**	**Blinding**	**Meeting attendance**^**c**^			
		**TMG**	**DMC (o)**	**DMC (c)**	**TSC**
**Model 1—Trial statistician can be unblinded**^**b**^ **to maintain blind of lead statistician**					
Trial statistician	Blinded at outset but can be unblinded to preserve blinding of lead statistician	(✓)	✓	✓	(✓)
Lead statistician	Remains blinded until database lock	✓	✓		✓
**Model 2—TS remains blinded, lead statistician (LS) can be unblinded**^**b**^					
Trial statistician	Remains blinded until database lock.	✓	✓		✓
Lead statistician	May become unblinded (if required) to maintain the TS blind. If not previously unblinded, they are unblinded at database lock	(✓)	✓	✓	(✓)
**Model 3—TS and LS remain blinded by having a third unblinded statistician**					
Trial statistician	Remains blinded until database lock	✓	✓		✓
Lead statistician	Remains blinded until database lock	✓	✓		✓
Non-blinded Statistician	Unblinded			(✓)	
**Model 4—TS and LS remain ‘pseudo-blinded’ by using coded groups**^**d**^					
Trial statistician	Remains “blinded” to true treatment allocations until database lock	(✓)	✓	✓	(✓)
Lead statistician	Remains “blinded” to true treatment allocations until database lock	(✓)	✓	✓	(✓)
**Model 5—Both statisticians can be unblinded**^**b**^ **during the trial**					
Trial statistician	May become unblinded if required. If not during the trial, they are unblinded at database lock	(✓)	✓	✓	(✓)
Lead statistician	May become unblinded if required. If not during the trial, they are unblinded at database lock	(✓)	✓	✓	(✓)
**Model 6—All statisticians are unblinded**^**b**^					
Trial statistician	Unblinded	(✓)	✓	✓	(✓)
Lead statistician	Unblinded	(✓)	✓	✓	(✓)

*CRF* case report form, *DMC* Data Monitoring Committee, *TMGs* Trial Management Groups, *TS* trial statistician, *TSC* Trial Steering Committee

^a^The focus groups also identified that in some instances the lead statistician (also referred to as senior or principal statistician) would delegate responsibility for certain tasks to another statistician (e.g. validation of statistical programming)

^b^Note SAP is written and signed off early (amendments recorded) so unblinded data can be seen

^c^Attendance at meetings: TMGs, DMC (open and closed), TSC (✓—Yes, (✓) —Sometimes))

^d^Coded allocation groups can be used to facilitate analysis and reporting of data split by treatment group without directly revealing participants’ allocated treatment

An undesirable feature of some of these models is the incomplete oversight that the lead statistician (LS) has over the TS. It is worthy of note that a benefit of models 5 and 6 (where the involvement of the TS and the LS match) limits the possibility of partial oversight of the TS.

Based on the participants’ views, Table 3 summarises the range of activities undertaken by CTU statisticians beyond the analysis and provides a brief description of each. These activities taken from focus groups and not an exhaustive list.

**Table 3:** Activities undertaken by CTU statisticians in the design and conduct of clinical trials

Activity	Description
Co-applicant	• A lead (or principal/senior) statistician who is involved in the grant application • A TS who is involved in the grant application
Trial oversight/reports^a^	• Attends TMGs, TSCs and DMCs as appropriate • Assists with report writing for trial management meetings and oversight committees (TSC and DMC)
Assists with trial processes^a^	• E.g. generating randomisation lists, dummy treatment allocations, data monitoring, data cleaning for example for missing data, data completion rates, etc. • Assist in the development and quality control (QC) of the clinical trial database and/or randomisation system • Level of engagement dependent on whether blinded or not
Assists in writing and reviewing core trial documents^a^	• E.g. protocol, risk assessment, CRF, SAP and data management plans • Assist with QC of core trial documents
Training/supervision	• A senior statistician takes on the oversight role for the TS (blinded or unblinded) • Provides peer or senior support, guidance and training to other statisticians
Analysis	• Conducts end of study analysis • Conducts any planned/unplanned interim analysis • Conducts parallel/double/dual coding of the primary outcome for interim or final analyses • Often the unblinded statistician will create dummy allocations.
Quality control^a^	• Validates/QCs any data prepared for reports, the final analysis or any interim analysis that lead to decision making

*CTU* clinical trials unit, *DMC* Data Monitoring Committee, *QC* Quality Control, *SAP* statistical analysis plan, *TMG* Trial Management Group*, TS* trial statistician, *TSC* Trial Steering Committee

^a^Some FG participants, both statisticians and other roles, stated that both data manager and programmer roles were also involved in activities such as treatment allocation and preparing unblinded reports for oversight committees

It is crucial to highlight that participants used a variety of terminology to describe the role of statisticians in RCTs, and therefore, it was important to establish an agreed nomenclature for the roles. For example, some participants were not sure what was meant by ‘independent statistician’ as these are not always truly independent (they often work for the same unit, just not on the study all the time). Some participants agreed that it might be simpler to use the terms ‘blind’ and ‘unblind’ to describe the statisticians.

### Theme 2—Factors affecting the decision to blind statisticians

#### Study design

Study design was an influential factor for most participants regarding whether to blind the statistician. For example, adaptive trial designs typically rely on interim outcome analysis, so to maintain blinding, these study designs may need more statisticians.

“… a separate DMC with a blinded interim analysis and keep them away from the TSC to see the data…, what we’re basing it on was the design of the trial and whether there is any consequences clinically.” (FG1, Trial manager)

A common view amongst participants was that if the trial was fully blinded (e.g. placebo controlled), this required a blinded/independent statistician, but that if the trial was not blinded (e.g. open label) then there was no need to blind the statistician.

“…I think it’s more important to consider blinding if it’s a fully blinded, you know, double-blind trial.” (FG6, Team leader)

A further common view was that the TS was more likely to be blinded for Clinical Trials of Investigational Medicinal Products (CTIMPs) due to the (MHRA) monitoring and frequent auditing.

“…if we did have to unblind the statistician in a non-CTIMP, we would maybe do that a little bit…more easily than if we had to do it for a CTIMP, but still I think we would try and adhere to the strict principles...” (FG3, Statistician)

#### Types of intervention

An intervention that was expected to cause harm or serious side effects was amongst the factors that most strongly influenced the decision to blind the statistician, whereas lack of blinding in the assessment of low risk interventions was considered less problematic by participants.

“… we knew that there were safety concerns with these particular participants, so don’t know if that would come into the decision to keep statisticians unblind or blind.” (FG4, Data manager)

#### Types of outcome

The majority of participants indicated that ‘type of outcome’ including adherence data, side effects and complications influenced their decision to blind the TS.

“…it’s very difficult to blind someone properly when they’re actually looking at all the data, because they may see things about treatment adherence and things which reveal the groups.” (FG2, Statistician)

Objective as opposed to subjective outcomes, e.g. death, blood markers, were also deemed less important to blind the TS as these were considered less open to interpretation, thereby reducing the risk of introducing bias.

“I think as a statistician you know the kind of endpoints that we are dealing with, the very hard endpoints is all quantified stuff that’s not open to interpretation…, so I don’t really see there’s a risk there.” (FG2, Statistician)

Most participants felt the issue as to who should be blinded in a trial to reduce bias was a wider issue than just the statisticians for example, recruiting staff, assessors collecting endpoints, and other staff assessing compliance of the trial protocol.

“Yeah, I also tend to agree that we should try to blind as much as possible, but I think the people to be targeted and the people who are targeted who are determining the eligibility of the patients, those who are evaluating the endpoints and those who are assessing their compliance of the protocol…” (FG1, Statistician)

#### Types of analysis

Whether there was a planned interim analysis influenced participant’s decision to allocate a blinded statistician. One participant said

“…if you’ve got a formal interim analysis partway through the trial, then it would be more important to have a blinded statistician because they have more insight into the results as you’re going along.” (FG4, Statistician)

Notably, all participants felt the statistician, blinded or otherwise should have a good knowledge of the clinical topic and intervention in order to fulfil their duties.

“…and a big part of the analysis that we’re doing is all about the treatment deliverability, and you know we’re calculating all the drug intensities or whatever. So you need to know which arm people are on, and it is crucial that the statistician understands and analyses that level of stuff.” (FG2, Statistician)

#### External factors

Various external factors were also highlighted by participants affecting the decision to blind the statistician(s). Participants indicated that funders often ask for blinded trials but not blinded statisticians. Although there are examples for trials where funders paid for multiple statisticians [16, 17], the perception and indeed experience of some participants was that funders pushed back on applications including funding for multiple statisticians.

“…funders don’t want to pay for multiple statisticians, as we know. I suspect the seniors have all had these arguments with funders even to get a senior statistician at a reasonable amount and a trial statistician. That’s where it by and large stops, and we can try to some extent hide additional resource within costings. But you know it’s very difficult to get that transparently...” (FG4, Statistician)

A minority mentioned that sponsors could influence the decision to blind the TS especially in CTIMPs trials, while almost all agreed that governing agencies, e.g. the MHRA in the UK or the FDA (Food and Drug Administration) in the USA, influenced decision-making.

“If it’s a CTIMP study it’s something they [sponsor] want, extra level security, so I think I’ve known at least one instance where it’s been kind of forced upon somebody when they really didn’t think it was in there, they were actually resistant to it but the sponsor was insisting on it….” (FG2, Statistician)

“I think the design’s a lot more robust as well…it’s going to be potentially monitored or you know, seen by the MHRA.” (FG6, Trial manager)

### Theme 3—Benefits of blinding versus not blinding statisticians

#### Benefits of blinding

The participants’ views on the reasons for blinding the TS were mainly focused on reducing the possibility of introducing conscious or unconscious bias.

Participants highlighted that having a blinded TS can contribute to the credibility and quality of the trial despite the risk of bias being low.

“…trials are always gonna be open to bias at a site level, within a CTU and industry …and I think with anything even if you don’t think there’s any inherent risk of not having blinded statisticians, then it gives a trial credibility doesn’t it just next to having that level of blinding within a trial.” (FG1, Trial manager)

Some participants also argued that a blinded TS would guarantee confidentiality by being unable to share data with staff who could bias the future results, such as TMG members, treating clinicians and outcome assessors.

“…having someone within this Stat's team that hasn't seen the allocations that the DMC and probably gives more of the rest of the team that's having the conversation a bit more confident that that isn't coming from someone that's seen the analysis as well.” (FG2, Statistician)

For some participants, reducing pressure on the statistician to reveal data if they were unblinded, thus, obviating the risk of influencing recruitment by talking and inadvertently providing information to the principal investigator (PI), were reasons for blinding TSs.

“…it might be more indirectly there is a leak of information somehow. It could then plausibly affect the recruitment process, so is it only at one site or something you’re talking to the PI and they may basically change who they recruit….” (FG2, Statistician)

#### Benefits of not blinding

The majority of participants believed that an unblinded TS, having a better understanding of the data in its context, would be able to contribute more meaningful input to the trial, for example when it came to clinical and safety decisions likely enhance the quality of the data analysis.

“…the idea that somebody comes in independent of the study halfway through and does some analysis is, I think can only have been dreamt up by someone who isn’t a statistician and doesn’t understand that to do a good analysis you have to understand the context of your data.” (FG5, Statistician)

Other benefits indicated by participants included having open conversations that might reduce the risk of incorrect decision-making and wrong assumptions about the data, as well as allowing the statistician to fully interrogate the data throughout the trial (e.g. estimate standard deviation in each group, which may impact on the primary analysis.) Another benefit was that an unblinded statistician was best placed to support the DMC in providing effective oversight of the trial.

“…the relationship between the trial statistician and the DMC committees is really important to provide that really good service so they can then make, you know good decisions on behalf of patients really, so I think that’s the main priority I think as opposed to kind of blinding.” (FG2, Statistician)

### Theme 4—Practicalities

How to maintain the blind of the statistician during trial delivery was a challenging factor raised by participants across all the FGs. Suggestions how this could be achieved included (1) putting in place rigorous processes enabling the blinded statistician to access and request data or allocation, (2) giving the responsibility to data management to prepare reports and strip out data which could unblind and (3) allowing the programmer to write and produce the randomisation system in order to ensure that no other team members have directly seen the allocation.

“…it does seem to me like there’s an awful lot of effort sometimes put into this that probably is not worthwhile. However, it does seem on the face of it like this would be a noble thing to do, even though you know the practicalities of it in many cases, you know, it’s an ideal which may not be quite properly attained in the end.” (FG1, Data manager)

Some participants stated that issues of staffing and unit capacity were factors that might influence the practicality of blinding the TS. Some participants questioned the reliability of the blinding process and whether the blinded statistician could be truly ‘blinded’ when they, for instance, sit next to the blinded TS in the same office.

“I mean I work in a blinded study and it’s difficult, you're always thinking of what not to say to people,…. So, you see someone that you work with day-to-day, that becomes even more difficult because you’re not used to censoring yourself within your team, I think that would be a risk of doing it. I don’t know how you negate that risk within a team.” (FG6, Statistician)

The level of statisticians’ experience and knowledge about blinding was also found to influence the decision whether to blind the TS, as well as whether other trial staff were blinded. Several participants felt a lack of training or awareness could lead to unblinding, for example by an unblinded member of the trial team sending information about allocation to the blinded statistician.

“there are resource implications and training really just training people why it is important, just like you say, bias never sleeps.” (FG1, Statistician)

Moreover, lack of experience was seen as a concern when

1. The blinded TS was unable to answer questions from the DMC to aid in their recommendations
2. They were not sufficiently skilled to run interim analysis
3. They were unable to recognise issues in the code where data do not make sense

“I started working as an [blinded] statistician on one of the trials and…I got really stuck, I just didn’t know what to suggest because I didn’t have very much experience then and I kind of wanted that second opinion of ‘what do you think is going on here’ … so I think that is an issue, especially if someone doesn’t have very much experience in that role, just you have to be confident in your ability to just liaise with the DMC and things like that.” (FG3, Statistician)

Some participants suggested that a minimum of four statisticians (senior statistician, unblinded-TS and two blinded-TSs) would be required to enable them to allocate blinded statisticians effectively. However, the availability of funding to the CTUs represented in the FGs, to secure the number of statisticians for each trial was considered impractical and unrealistic, along with challenges in recruitment.

“The other problem is the restrictions put on units by the funders that they will only kind of accept up to a certain amount of time for an analysing statistician and the knock-on implications for resource then quite profound because what you ideally want probably is twice the amount of statistician time but realistically, I don't think, you're likely to get that.” (FG6, Statistician)

There was general agreement between participants it should be recommended to engage TSC/DMC committees as early as possible to understand the extent of disaggregated data they would like to see during the trial. For example, the DMC will usually consider safety data by treatment group as a minimum but in some cases might not want to compare outcome data by treatment group. Safety listings might not require unblinding of the statistician while analysis of outcome data will.

“Sometimes it depends on what the DMC requests. So we might start off being blinded…but the DMC were like, no we want to know, so then I was aware of treatment allocation.” (FG4, Statistician)

Nearly all participants indicated the need to include blinding as part of a risk assessment and use that to derive what level of TS blinding was needed in a given trial, based on the context.

“…needs to be part of this sort of risk assessment process at the start of the trial, and that might help us manage some of the diversity we’re always going to have to contend with across units and in the [CTU] network.” (FG4, Unit director)

## Discussion

This study found substantial variation in practice between CTUs with respect to blinding the TS because of various factors such as study design, types of interventions, types of outcomes and available funding as well as different opinions on the importance and feasibility of blinding. Therefore, the most important finding is that a ‘one size fits all’ approach is not practical in the process of deciding when/how the TS should be blinded.

### Statistical models of work

Although there were varying opinions on when and whom to blind in regard to statisticians, all models shared that there are at least two statisticians involved, one more junior (trial statistician) and one more senior (lead/principal statistician). The findings suggest that blinding is at least attempted in most scenarios despite resource restrictions, with the option to unblind one or both statisticians if necessitated.

Only one model included a formal third statistician to take on a purely unblinded role in order to maintain the blind of the TS and senior statistician. This model is consistent with the recommendation made within MHRA Good Clinical Practice guidance for Clinical Trials of Medicinal Products (CTIMPs) for human use in the UK [9]. However, there is currently no guidance on blinding for trials of non-CTIMPs.

Although prior studies have noted the importance of keeping investigators and sponsors blinded to interim analysis data in order not to endanger the successful completion of a trial [18, 19], the role of the study statistician in this regard has not been thoroughly addressed. In this study, participants felt that statisticians are unlikely to introduce bias via their influence on the conduct of the trial. In particular, it was suggested that the benefits of blinding the statistician might not always outweigh the drawbacks. They nearly all agreed that the insight and existing knowledge and skills the statistician brings to the table is paramount, and so it is vitally important that blinding does not create an impediment to this. This view accords with other studies which showed that the risk to the trial of an unblinded TS disclosing interim results is extremely low and that the use of a third, independent statistician could introduce disadvantages outweighing the potential benefits for a given trial [15, 20].

### Factors affecting the decision to blind statisticians

Our findings suggest that the trial characteristics should be used more effectively to help the decision process whether to blind the statistician(s). It is interesting to note that in all FGs most participants felt that it was redundant to blind statisticians in an open label trial where recruiting staff, clinicians, and outcome assessors are unblinded. However, there should be care not to equate blinding of trial statisticians with other members of the research team, as statisticians will typically also have access to accumulating outcome data for all participants.

Another important factor described by our participants was the study design where they suggested that the decision to blind the statistician would be influenced by trials that have complex or adaptive designs. A recent study showed that observing data at each interim analysis in adaptive studies requires careful considerations in order to eliminate the risk of bias and ensure the trial’s integrity by keeping investigators and other people with a vested interest in the study blinded wherever possible [21].

There are similarities between the views expressed in this study and those described in the literature which suggest that blinding during a superiority trial is more important than in a non-inferiority trial, e.g. in order to avoid the investigators from manipulating the results to support their beliefs [22].

### Benefits of blinding versus not blinding statisticians

Blinding statisticians was considered beneficial in reducing the risk of biasing outcomes by revealing data to other research teams who have a direct impact on study recruitment or assessment of the effectiveness of interventions.

Although blinding is generally viewed as an effective method by which to mitigate bias [23], blinding poses certain limitations, and there are some profound benefits of unblinding certain members of the research team including statisticians [24]. Thus, there are still questions about how tangible is ‘bias’ and is experience and knowledge to assist a trial more important than blinding to reduce perception of ‘bias’?

The main benefits of not blinding statisticians identified were the greater insight afforded the statistician from their understanding of the data and its context, which leads to higher quality analysis, and decreasing the risk of incorrect decision-making and wrong assumptions about the data. These results are in line with those of advocates of pragmatic studies who found blinding statisticians to be a deviation from real-world experience [25] which may degrade the quality of trial monitoring and will not guarantee the validity of the conclusions [20].

It is imperative to stress that our work was focused on blinding statisticians and most of the evidence used to compare with other studies was concerned with blinding staff other than statisticians, e.g. clinicians, participants, investigators and outcome assessors.

Of course, given sufficient resources, one can profit from the merits of both blinded and non-blinded trial statisticians; however, the resource required to have multiple statisticians sufficiently immersed within a trial should not be underestimated, and is seldom attained within the setting of UK academic CTUs.

### Practicalities

Although there was some agreement on how blinding of the statistician(s) could be achieved, it was typically deemed unrealistic and potentially unnecessary for all trials.

In RCTs, maintaining a study blind takes effort, planning and resource which in some cases can be considerable [26]. This was reflected in our findings in the description of the wide range of activities statisticians are involved in throughout the lifespan of a trial [25] as well as reference to the lack of funding. However, the current deficits in the pipeline of statistical expertise were one of the main concerns emerging from this study. Engaging with experienced statisticians can help to avoid unworkable blinding options, as well as ensuring that the work required, e.g. such aspects as running interim analysis and preparing DMC reports, is delivered efficiently and on time.

There were similarities between the attitudes expressed by the participants and those described by Karanicolas et al. [1], about best practice being to blind as many individuals as possible in order to minimise the risk of differential assessments of outcomes in a trial [1].

Consistent with the significant heterogeneity characterising this study’s findings in respect of “when” and “how” to blind statisticians, it is important to appreciate the importance of developing a risk assessment to enable CTUs to make informed proportionate decisions about what level of TS blinding is needed in a given trial, based on various factors identified in this study.

### Strengths and limitations

Strengths of this study include the approaches used to maintain scientific rigour and trustworthiness. First, methodologically, this is a novel study to explore blinding of statisticians in RCTs. Second, the study managed to achieve a broad understanding of various experiences of blinding statisticians by including multiple stakeholders from several CTUs across the UK. Third, two independent researchers, experienced in thematic analysis, conducted coding. The emerging codes were then discussed between the two researchers and a third team member (statistician), to ensure proper interpretation of the data and reliability of the results.

A strength of the study is also a limitation as this study is limited by the focus on data from UK academic CTUs which limits the findings’ generalisability. Although various stakeholders participated in the FGs, we appreciate that there were particular groups under-served in this study e.g. regulatory bodies and sponsors.

## Conclusion

To the best of our knowledge, this is the first study to shed light on the blinding status of statisticians in RCTs. Using focus group discussions, four main themes were identified:

(i) Models of work
(ii) Factors affecting the decision to blind/not blind statisticians
(iii) Benefits of blinding/not blinding
(iv) Practicalities and best practice

Although there was variation in practice, and pros and cons of both blinding and not blinding, two significant findings emerged from the study. First, where resources are limited, there is a tension between the twin aims of minimising the risk of bias and maximising the insight of statisticians to improve analysis, reporting and decision making. Second, variation in practice is inevitable because of numerous factors affecting each RCTs such as resources/staffing and study design. The findings from this study are now being used to develop evidence-based and risk-proportionate guidance and a risk assessment tool to guide CTUs with regard to blinding statisticians in RCTs.

## Supplementary Information

**Additional file 1.** Participant information sheet.

**Additional file 2.** Informed consent form.

**Additional file 3.** Topic guide.

**Additional file 4.** The consolidated criteria for reporting qualitative research (COREQ).

## Data Availability

Data are available on reasonable request. The unpublished data used and/or analysed during the current study are available from the corresponding author on reasonable request.

## Abbreviations

BOTS: Blinding of trial statisticians
CI: Chief investigator
CRF: Case report form
CTIMPs: Clinical Trials of Investigational Medicinal Products
CTU: Clinical trials unit
DMC: Data Monitoring Committee
DSMC: Data and Safety Monitoring Committee
FDA: Food and Drug Administration
FG: Focus group
MHRA: Medicines and Healthcare products Regulatory Agency
MRC: Medical Research Council
NIHR: Institute for Health Research
PI: Principal investigator
QC: Quality control
RCT: Randomised clinical trial
SAP: Statistical analysis plan
TMG: Trial Management Group
TMRP: Trials Methodology Research Partnership
TS: Trial statistician
TSC: Trial Steering Committee
UKCRC: UK Clinical Research Collaboration
UKTMN: UK Trial Managers’ Network
