# Supplementary material for: Blinding of study statisticians in clinical trials: a qualitative study in UK clinical trials units
Source: Trials. 2022 Jun 27;23:535. doi: 10.1186/s13063-022-06481-9 (PMC9235168; doi:10.1186/s13063-022-06481-9)
Supplement: Supplementary file 1 — Additional file 1. Participant information sheet. [file 13063_2022_6481_MOESM1_ESM.docx]

**Additional file 1: Participant Information Sheet**

**Study title: Blinding of the trial statistician in clinical trials**

We would like to invite you to take part in an interview/focus group to talk about and reflect on your experience of blinding of trial statisticians who work in the delivery and oversight of clinical trials.

**What is the purpose of this study?**

There is good empirical evidence of the benefit of blinding participants, doctors and researchers in clinical trials to reduce the risk of bias and improve the trust and credibility of the results. However, it is not clear if blinding of other members of the trial team, such as statisticians, also reduces the risk of bias. Existing guidelines recommend blinding the trial statisticians before the end of the trial, but do not consider the trial-specific risk of blinding or not blinding the statistician. The goal of this project is to create guidance for if and when blinding statisticians is appropriate, that is related to the risks associated with a given trial.

**What does the study involve?**

We wish to explore the experiences, opinions and ideas of key stakeholders on blinding of trial statisticians who work in the delivery and oversight of clinical trials. We would like to engage a wide range of stakeholders including Statisticians, Clinical Trials Unit (CTU) directors, Data Monitoring Committee (DMC) chairs, Trial Managers, Data Managers and Unit Managers.

If you agree to participate, you will be asked to attend a focus group, however, if this is not feasible, we may ask you to join an individual interview.

*Focus group*

A focus group is an organised discussion with a group of people to gain their views and experience in a specific topic, which in our study will be the experiences, opinions and ideas on blinding of trial statisticians. You will join a focus group with other participants. The researcher(s) will contact you to arrange a suitable time for a focus group. The focus group will be conducted using the Microsoft Teams Platform, will be video and audio recorded and will last approximately 90 minutes.

*Individual interview*

The researcher(s) may conduct individual interviews with some stakeholders, where focus groups might not be convenient due to time constrains. The purpose of these interviews is to explore participant's perceptions of blinding of trial statisticians. The interview will be conducted using the Microsoft Teams Platform, will be video and audio recorded and will last approximately 60 minutes.

- The questions in both focus groups and interviews will be open-ended, to allow you the chance to raise the issues that you feel are important.

**Do I have to take part?**

Taking part is entirely voluntary but if you do take part you can withdraw from the study at any time. If you decide to participate, you will be asked to give consent.

**Are there benefits to me in taking part in this study?**

Sharing your experiences and ideas will help us to develop a guidance document on blinding of trial statisticians and any resulting recommendations for change.

**Will what I say be confidential?**

Yes. We will follow ethical and legal practice and all information about you will be handled in confidence. The data will be anonymised by using codes on focus group and interview transcripts. Any quotes used in the research, e.g. publications, presentations at conferences or seminars will use non-identifiable codes rather than the participant’s name. Electronic data will be password-protected and saved on the University of Nottingham’s server.

University of Nottingham will keep non-identifiable information about you for seven years after the study has finished.

All focus group participants will be reminded not to repeat what is discussed to others outside the group.

The research team will use your name, email, and telephone number to contact you about the research study. The only people in the University of Nottingham who will have access to information that identifies you will be the research team who need to contact you to arrange for focus groups and interviews. Your contact details will be deleted as soon as possible after the interview or focus group has taken place and is no longer needed.

**What will happen to the results of the study?**

Once the focus groups and interviews has been transcribed and analysed, the results will be used to assist in developing a guidance document to advise CTUs on a risk proportionate approach to blinding statisticians within trials. Findings will be published in scientific journals and presented in conferences, seminars and workshops. Quotes from focus groups and interviews may be used, but will be anonymised.

**Who is organising and funding the study?**

The study is being led by researchers from the Nottingham Clinical Trials Unit, University of Nottingham and funded by the NIHR CTU Support Funding.

**Who has reviewed the study?**

The study has been reviewed and approved by the University of Nottingham Research Ethics Committee (March 26, 2021).

**What do I do if I have a concern about the conduct of this study?**

If you have any concerns about any aspect of the study, you should contact the Chief Investigator, Dr Christopher Partlett or the researchers, Kirsty Sprange or Mais Iflaifel.

**Where can I get more information?**

If you have any questions, please contact:

- Dr Christopher Partlett, Chief Investigator. Email: [chris.partlett@nottingham.ac.uk](mailto:%20chris.partlett@nottingham.ac.uk)
- Kirsty Sprange, Co-investigator. Email: [kirsty.sprange@nottingham.ac.uk](mailto:kirsty.sprange@nottingham.ac.uk)
- Mais Iflaifel, Co-investigator. Email: [mais.iflaifel@nottingham.ac.uk](mailto:mais.iflaifel@nottingham.ac.uk)
