# Supplementary material for: Blinding of study statisticians in clinical trials: a qualitative study in UK clinical trials units
Source: Trials. 2022 Jun 27;23:535. doi: 10.1186/s13063-022-06481-9 (PMC9235168; doi:10.1186/s13063-022-06481-9)
Supplement: Supplementary file 2 — Additional file 2. Informed consent form. [file 13063_2022_6481_MOESM2_ESM.docx]

**Chief Investigator**

Dr Christopher Partlett

[chris.partlett@nottingham.ac.uk](mailto:%20chris.partlett@nottingham.ac.uk)

**Co-investigators**

Kirsty Sprange

[kirsty.sprange@nottingham.ac.uk](mailto:kirsty.sprange@nottingham.ac.uk)

Mais Iflaifel

[mais.iflaifel@nottingham.ac.uk](mailto:mais.iflaifel@nottingham.ac.uk)

**Additional file 2: Informed Consent Form**

**Study title: Blinding of the trial statistician in clinical trials**

1. I confirm that I have read and understand the Participant Information Sheet dated ______________for the above study. I have had the opportunity to consider the information, ask questions and have had these answered satisfactorily.
2. I understand that my participation is voluntary and that I am free to withdraw at any time.
3. I understand and give permission for the focus group/interview to be audio and video recorded and for the resulting data to be used in reports and publications including anonymous direct quotation.
4. I understand that data collected will be stored securely, in accordance with the General Data Protection Regulation 2018 (GDPR) and the Data Protection Act 2018 which controls how your personal information is used by any organisation.
5. I understand that I am not obliged to answer any question in the focus group or interview.
6. I understand that I will not reveal any information that is shared in confidence in the focus group.
7. I understand that the data collected from me in this study will be preserved and made available in anonymised form, so that they can be consulted and re-used by others.
8. I agree to take part in the above study, which has been subject to ethical reviews according to the procedures specified by the University of Nottingham Research Ethics Committee (March 26, 2021).

**Participant details**

Name of Participant: ­­­­**_______________________________________**

Signature: Date:

**Witnessed by**

Name of researcher taking consent:

Signature: Date:
