# Supplementary material for: Blinding of study statisticians in clinical trials: a qualitative study in UK clinical trials units
Source: Trials. 2022 Jun 27;23:535. doi: 10.1186/s13063-022-06481-9 (PMC9235168; doi:10.1186/s13063-022-06481-9)
Supplement: Supplementary file 3 — Additional file 3. Topic guide. [file 13063_2022_6481_MOESM3_ESM.docx]

**Additional file 3: Topic Guide**

1. **Welcome**

- I am MI, a researcher from Nottingham Clinical Trials Unit, University of Nottingham, conducting a research study about blinding trial statisticians in clinical trials.
- Thank you for agreeing to participate. The aim of the focus group/interview is to explore in depth your experiences, opinions, and ideas on blinding of trial statisticians. We are keen to learn from you, as your input will help us developing evidence-based guidance and recommendations on the practice of blinding statisticians.

1. **Focus group/interview ground rules**

- Before we start, I would like to mention a few things about this meeting.
- To maintain confidentiality, I would ask that information provided in the focus group must be kept confidential by the focus group participants. Please do not discuss or share with others beyond this group, what is said in this meeting. (Focus group)
- The research team will handle confidentiality of all identifiable information (names, email and/or telephone numbers, voices). (Focus group and interview)
- It is important for us to hear each other, contribute in the discussion. Please can one person speak at any one time. (Focus group)
- If it is at all possible, please could all mobile phones be switched off or turn to silent. I anticipate this focus group discussion to last for approximately 90 minutes. (Focus group and interview)

Just to check if everyone happy to turn the recorder on.

1. **Turn on the recorder**

Could everyone please introduce themselves and their role.

Let’s begin:

**Definitions**

*Blinded statistician: Statistician with no access to data regarding treatment groups, including coded group names (e.g group A, group B). This also includes any information that may be used to indirectly infer treatment allocation.*

*Interim analysis: Any analysis prior to the final analysis involving disaggregate data (that is, data analysed by treatment group – even if only in coded format (e.g group A, group B).*

| 1. There are various models and different roles that statisticians can undertake in randomised trials. Could you explain what these models/roles are and specifically how each role is involved in the design and delivery of randomised trials?   (Prompt: Senior, junior, oversight, independent and trial statistician) |
| --- |
| 1. Why do you think it is important to blind statisticians? What are the benefits?   (Prompt: credibility, robustness, ethical, evidence, influencing outcomes, bias, protection for the statistician)   1. If we asked the question in a different way? What are the consequences (benefits or drawbacks) of not blinding statisticians?   (Prompt: understanding data in context) |
| 1. Expanding more on the previous point, in your experience when and how each role should be blinded/not blinded?   (Prompt: other team are unblinded, is it practical in real life to keep statisticians blinded, is it taken seriously?) |
| 1. What factors might affect the decision to blind/ or not blind statisticians?   For instance:  Types of study design  Types of intervention  Types of outcome  Types of analysis (e.g. per protocol etc.)  Resources (e.g. cost, recruitment, skills, funding sources, local partner support)  Expectations (e.g. funders, sponsors, industry, other stakeholders)  Processes (e.g. SAP, SOPs, QA, historical/new processes)  Logistics (e.g. unit size, stats team size) |
| 1. Which of these factors shown on the screen, do you feel most important to include in a risk proportionate approach to blind/not blind statisticians? 2. Could I ask everyone to use the link in the chat box to participate in a quick survey about blinding statisticians. |
