# Supplementary material for: Blinding of study statisticians in clinical trials: a qualitative study in UK clinical trials units
Source: Trials. 2022 Jun 27;23:535. doi: 10.1186/s13063-022-06481-9 (PMC9235168; doi:10.1186/s13063-022-06481-9)
Supplement: Supplementary file 4 — Additional file 4. The consolidated criteria for reporting qualitative research (COREQ). [file 13063_2022_6481_MOESM4_ESM.docx]

**Additional file 4: The consolidated criteria for reporting qualitative research (COREQ)**

| **Domain 1: Research team and reflexivity** |  | | Location in manuscript (Section, page no.) | |
| --- | --- | --- | --- | --- |
| **Personal Characteristics** | | | | |
| 1. Interviewer/facilitator Which author/s conducted the interview or focus group? | MI, CP and KS | | Recruitment and data collection, 3. | |
| 2. Credentials  What were the researcher’s credentials? E.g. PhD, MD | CP-PhD  MI-PhD  KS- | | - | |
| 3. Occupation  What was their occupation at the time of the study? | CP- Assistant Professor of Medical Statistics and Clinical Trials  MI- Research Fellow  KS- Assistant Professor | | - | |
| 4. Gender Was the researcher male or female? | Two females and one male | | - | |
| 5. Experience and training  What experience or training did the researcher have? | At the time of the focus groups, both MI and KS had a long experience in qualitative methods and CP has extensive experience in statistics in clinical trials | | - | |
| **Relationship with participants** | | | | |
| 6. Relationship established  Was a relationship established prior to study commencement? | Yes | | - | |
| 7. Participant knowledge of the interviewer  What did the participants know about the researcher? e.g. personal goals, reasons for doing the research | Participants were briefed on the purpose of the study and understood that aim was to explore their perspectives about blinding statisticians in clinical trials. Ethical approval had been granted, participants reviewed the participant information sheet and provided their informed consent before starting the focus group. | | Methods- 2-3. | |
| 8. Interviewer characteristics What characteristics were reported about the interviewer/facilitator? e.g. Bias, assumptions, reasons and interests in the research topic | CP is a statistician who might be a potential source of bias. However, CP was only facilitating without introducing ideas or opinion that could influence other participants’ perspectives. No other interviewer-related biases identified. | | Discussion – 15. | |
| **Domain 2: study design** | | | | |
| **Theoretical framework** | | | | |
| 9. Methodological orientation and Theory  What methodological orientation was stated to underpin the study? e.g. grounded theory, discourse analysis, ethnography, phenomenology, content analysis | Inductive and deductive coding with thematic analysis. | | Methods – 2. | |
| Participant selection | | | | |
| 10. Sampling  How were participants selected? e.g. | Recruited via email | | Methods – 3. | |
| 11. Method of approach How were participants approached? e.g. face-to-face, telephone, mail, email | Email | | Methods- 3. | |
| 12. Sample size How many participants were in the study? | 37 | | Results - 4 | |
| 13. Non-participation How many people refused to participate or dropped out? Reasons? | Of the 37 respondents that were invited for focus groups, only one participant was unable to participate and suggested a colleague name to join the focus group. No participant withdrew consent or dropped out. | | - | |
| **Setting** | | | | |
| 14. Setting of data collection  Where was the data collected? e.g. home, clinic, workplace | | Data was collected via Teams platform. | | Methods- 4 |
| 15. Presence of non-participants  Was anyone else present besides the participants and researchers? | | No | | - |
| 16. Description of sample  What are the important characteristics of the sample? e.g. demographic data, date | |  | | Table 1- 4. |
| **Data collection** | | | | |
| 17. Interview guide Were questions, prompts, guides provided by the authors? Was it pilot tested? | | We used a topic guide to facilitate focus group discussions. (Additional file 3) | | Methods – 2-4. |
| 18. Repeat interviews  Were repeat interviews carried out? If yes, how many? | | No | | - |
| 19. Audio/visual recording  Did the research use audio or visual recording to collect the data? | | The focus groups were video and audio recorded. | | Methods – 2-4. |
| 20. Field notes  Were field notes made during and/or after the interview or focus group? | | Field notes were also used to record discussions and agreement during FGs. | | Methods – 2-4. |
| 21. Duration What was the duration of the interviews or focus group? | | The focus groups duration was between 65- 90 minutes | | Methods – 2-4. |
| 22. Data saturation  Was data saturation discussed? | | No | | - |
| 23. Transcripts returned Were transcripts returned to participants for comment and/or correction? | | No | | - |
| **Domain 3: analysis and findings** | | | | |
| **Data analysis** | | | | |
| 24. Number of data coders  How many data coders coded the data? | | Two | | Methods- 2-3. |
| 25. Description of the coding tree  Did authors provide a description of the coding tree? | |  | | - |
| 26. Derivation of themes  Were themes identified in advance or derived from the data? | | Themes were derived from the data | | Methods – 2-3. |
| 27. Software What software, if applicable, was used to manage the data? | | Nvivo | | Analysis - 3 |
| 28. Participant checking  Did participants provide feedback on the findings? | | No | | - |
| **Reporting** | | | | |
| 29. Quotations presented Were participant quotations presented to illustrate the themes / findings? Was each  quotation identified? e.g. participant number | | Yes, specific comments were supported with direct quotes attributed to anonymised participant by role and focus group number | | Results – 5-14. |
| 30. Data and findings consistent  Was there consistency between the data presented and the findings? | | Yes | | - |
| 31. Clarity of major themes  Were major themes clearly presented in the findings? | | Yes | | - |
| 32. Clarity of minor themes  Is there a description of diverse cases or discussion of minor themes? | | No | | - |
